# Case Report: Young Adults With Breast Cancer: A Case Series of Fertility Preservation Management and Literature Review

**DOI:** 10.3389/fmed.2021.670872

**Published:** 2021-08-06

**Authors:** Mohd Faizal Ahmad, Yodo Sugishita, Yuki Suzuki-Takahashi, Shino Sawada, Hideyuki Iwahata, Eriko Shiraishi, Seido Takae, Yuki Horage-Okutsu, Nao Suzuki

**Affiliations:** ^1^Department of Obstetrics and Gynecology, St. Marianna University School of Medicine, Kawasaki, Japan; ^2^Department of Obstetrics and Gynaecology, Universiti Kebangsaan Malaysia Medical Centre, Kuala Lumpur, Malaysia; ^3^Laboratory of Cancer and Reproductive Science, Department of Frontier Medicine, St. Marianna University, School of Medicine, Kawasaki, Japan; ^4^Department of Obstetrics and Gynecology, Jikei University School of Medicine, Tokyo, Japan

**Keywords:** breast cancer, cryopreservation, fertility preservation, oncofertility, young adult

## Abstract

Breast cancer comprised at least 21.8% of the overall cancer among young adult (YA) women and became the leading cancer in this group in Japan, with 50% adolescent and YAs being diagnosed and 15–44-year-old women showing excellent 5-year survival. Surgical-chemoradiation therapy often results in excellent survivorship with an increased incidence of treatment-induced subfertility. Therefore, adding fertility preservation (FP) to the primary cancer treatment is necessary. Herein, we reported a series of cases of YA women with breast cancer who opted for FP, where their option was tailored accordingly. To date, the selection of oocytes, embryos and ovarian tissue is widely available as an FP treatment. PGT could reduce the risk of BRCA mutation transmission amongst BRCA carriers before pregnancy planning. Otherwise, gonadotropin-releasing hormone analog has no gonadoprotective effect and thus should not be considered as an FP option.

## Introduction

Breast cancer comprised at least 21.8% of the overall cancer among young adult (YA) women and became the leading cancer in this group in Japan, followed by cervical cancer at 12.8% and malignant germ cells and other gonadal tumors at 8.5% (1). Overall, 50% of breast cancer cases were among adolescent and YAs (AYA); in addition, the age group of 15–44 years old showed an excellent 5-year survival rate of almost 90% in localized cancer group, 80% in regional cancer group, and 35% in distant metastasis cancer group (2). The combination of surgical and chemoradiation therapy in managing breast cancer often results in excellent survivorship. However, it could also lead to a reduction in fecundity. Therefore, considering the increased incidence of chemotherapy-induced subfertility that leads to a devastating quality of life, adding fertility preservation (FP) to the primary cancer treatment is deemed essential in oncofertility services (3). A prompt strategy is paramount to evaluate FP's best option tailored to age, cancer stage age, and marital status. Herein, we reported a series of cases of YA women with breast cancer who opted for FP, where their option was tailored accordingly.

## Case Series

Our first case is a 31-year-old single and nulliparous woman with newly diagnosed stage I of right breast cancer (T1N0M0). Her hormone receptors were found positive (ER+/PR+) with HERS2-ve and Ki67 < 10%. Therefore, she was planned for right mastectomy and sentinel axillary lymph nodes biopsy, followed by possible chemotherapy (Taxane-based group if the nodes were positive). She was also planned for tamoxifen (TAM) therapy for at least 10 years. Therefore, she was referred to us for FP with an interval of 10 weeks before operation intervention. Her level of anti-Mullerian hormone (AMH) was 4.51 ng/dL. Given that she was single with applicable timeframe, a choice of oocyte cryopreservation was deemed appropriate. She was keen to start controlled ovarian stimulation (COS) as soon as possible; thus, the random start (RS) protocol with an aromatase inhibitor (AI) was offered. She managed to cryopreserve 10 oocytes and is currently still ongoing second COS before embarking on surgery next month. Although the chemotherapy is not yet planned, she was referred for possible long-term endocrine therapy for 10 years. By the time of treatment completion, the AMH could decline due to aging. Given that she has a borderline AMH level, the prediction of a further decrease in AMH level made the FP essential in managing her condition. However, oocyte cryopreservation could help motivate compliance to the primary treatment disease as her fertility ability has been covered.

Our second case is a 28-year-old married woman with preliminary diagnosis of left breast cancer upon tissue biopsy. Unfortunately, her lymph nodes tissue was found to be positive. Thus, she was categorized as T2N1M0. However, her hormonal subtype was ER/PR+. HERS2 was also positive and Ki67 > 20% (luminar B-like tumor). She was counseled for a left mastectomy with unilateral axillary lymph node clearance, followed by chemotherapy; anthracycline-based group or combination with taxane-based regime depending on the final histopathology examination and immunochemistry assessment of post-surgical specimen. She was also counseled regarding the possibility of anti-HER2 therapy for 1 year, followed by TAM for at least 5 years. Her current AMH level was 2.32 ng/dL. Therefore, she was counseled for FP treatment because the chemotherapeutic agent is gonadotoxic and long-term therapy because she is married. The option of embryo cryopreservation was an excellent choice. The interval before the primary cancer treatment was 6 weeks. Thus, adequate time was available for her FP. Fortunately, she was on her second day of menses; thus, conventional COS was initiated. To date, she had eight embryos cryopreserved (blastocyst stage) following two cycles of conventional COS. She is currently receiving chemotherapy and was planned for years for a cryopreservation update. She responded well with the chemotherapy (doxorubicin + cyclophosphamide), and she is currently on trastuzumab. Her latest AMH was 0.08 ng/dL. She experienced amenorrhea after 6 months of chemotherapy. Due to the FP strategy, she secured her chance for pregnancy in the future despite having a poor ovarian reserve due to her primary cancer treatment.

Our third case is a 37-year-old single and nulliparous woman diagnosed with right breast cancer 6 years ago. She was referred to us previously for FP treatment. She was diagnosed as triple negative because all her hormonal receptors were negative. However, her BRCA status was unknown due to financial constraints for testing. Her AMH level was 3.18 ng/dL. The timeframe for stimulation was limited at that time as her chemotherapy was scheduled a week after the FP counseling. Thus, the ovarian tissue cryopreservation (OTC) opted to follow FP counseling. She underwent laparoscopic left oophorectomy for OTC in November 2013. We obtained 23 pieces of ovarian tissue (1 mm^3^ per piece) and 13 MII oocytes after *in-vitro* maturation; both were cryopreserved. To date, she completed her chemotherapy (doxorubicin + cyclophosphamide and olaparib), and she is currently in remission. Her latest AMH was 0.96 ng/dL. Given that she is still single, she had no plan to fertilize the oocytes and continue follow-up yearly to update her cryopreservation status.

The last case is a 33-year-old nulliparous married woman with grade III intraductal carcinoma of the right breast, with ER/PR+, HERS2+, and Ki67 > 20%. Her current AMH level was 2.54 ng/dL. She was diagnosed in February 2018, and she underwent right mastectomy with ipsilateral axillary lymph node clearance a month later. She was referred to us within 6 weeks before the initiation of chemotherapy. We offered embryo cryopreservation but were only able to pursue a single COS cycle. She managed to preserve three good-quality embryos at that time (blastocyst stage). She received four cycles of doxorubicin and trastuzumab for 1 year and completed 3 years of TAM. Given that she is now in remission, she is keen to embark on pregnancy, with clearance obtained from her breast oncologist. She was planned for frozen embryo transfer (FET), with natural cycles for at least 3 months following the last dose of TAM as the “wash-out” period. Tentatively, her FET was planned for September 2021. Although the period was regular, her AMH level 6 months ago was 0.07 ng/dL, confirming the chemotherapy's gonadotoxic effect on her ovarian reserve. Thus, FP was an appropriate choice in her case.

All these cases represented the current scenario of managing breast cancer among YA women, where FP is deemed essential to be incorporated into the primary disease management. The gonadotoxic effect of the chemo-regime in breast cancer is contemplating, and recommendations seem to be inconclusive. Therefore, a proactive strategy in adding FP as a wise strategy in managing young women with breast cancer is appropriate. The summary of the cases is tabulated in Table 1.

**Table 1:** The summary of main characteristics and reproductive outcomes.

**Case**	**Background**	**Diagnosis**	**Treatment**	**FP Indication**	**FP Outcome**
1	31, Single Nulliparous	Right Breast Ca Stage I (T1N0M0) Luminar A Tumor ER+/PR+ HERS2-ve Ki67 < 10% AMH 4.51 ng/dL	*Chemotherapy*: Possible – Taxane-based group *Suppression Therapy*: Tamoxifen- 10 years	Long term endocrine therapy Possible of age related decrease ovarian reserve	Completed 1 COS-OC On dual stimulation – On-going 2nd Cycle 1st Cycle- 10 Oocytes Cryopreservation (OC)
2	28, Married, Nulliparous	Left Breast Ca Stage II (T2N1M0) Luminar B-Like Tumor ER+/PR+ HERS2 +ve Ki67 > 20% AMH 2.32 ng/dL	*Chemotherapy*: Doxorubicin, Cyclophosphamide *Suppression Therapy*: Trastuzumab-1 year, Tamoxifen- 5 years	Gonadotoxicity of chemotherapy Possible of age related decrease ovarian reserve	Completed 2 COS-IVF Embryo Cryopreservation (EC)- 8 Blastocyst AMH 0.08 ng/dL
3	37, Single, Nulliparous	Right Breast Ca ER–/PR HERS2 -ve (Triple Negative) AMH 3.18 ng/dL	*Chemotherapy:* Doxorubicin, Cyclophosphamide with Olaparib *Suppression Therapy*: -	Gonadotoxicity of chemotherapy Possible of age related decrease ovarian reserve	Ovarian Tissue Cryopreservation (OTC) (left oophorectomy)+ *in-vitro* Maturation (IVM) 13 Oocytes Cryopreservation (OC) 23 pieces of ovarian tissue AMH 0.96 ng/dL
4	33, Married, Nulliparous	Intraductal carcinoma grade III (IDC) Luminar B-Like Tumor ER+/PR+ HERS2 +ve Ki67 > 20% AMH 2.54 ng/dL	*Chemotherapy:* Doxorubicin *Suppression Therapy*: Trastuzumab-1 year, Tamoxifen- 3 years	Gonadotoxicity of chemotherapy Possible of age related decrease ovarian reserve	Completed 1 COS-IVF Embryo Cryopreservation (EC) - 3 Blastocyst AMH 0.07 ng/dL

## Discussion

**Figure 1:**
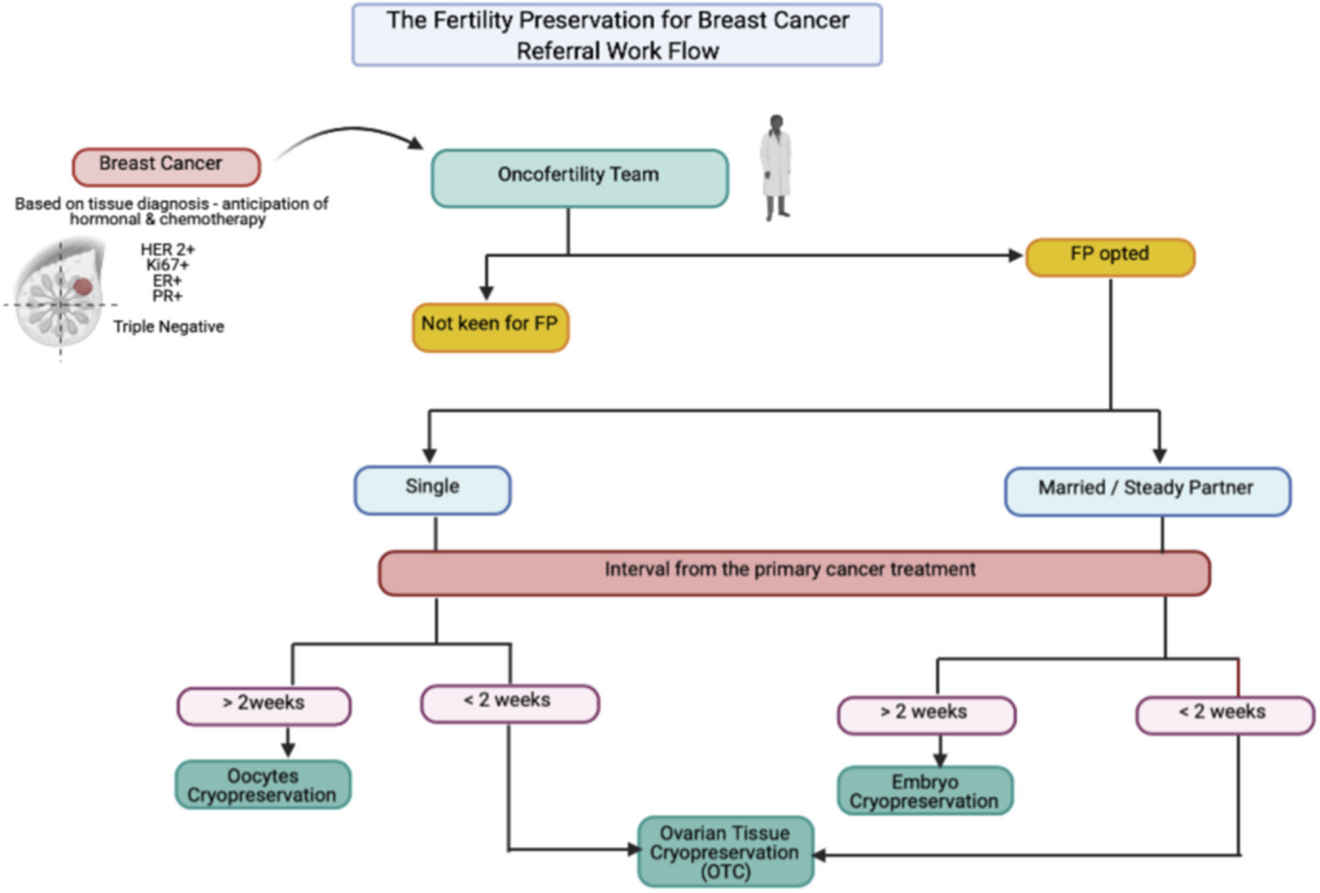
The suggested referral work flow for oncofertility referral for breast cancer cases.

As known, at least 15–25% of breast cancer cases affected premenopausal women, with 7% below 40 years old, which is categorized as AYA group (3). This group usually has a poorer prognosis than the post-menopausal group. However, the surviving rate increased by up to 80–90% in locoregional type due to the efficient breast cancer treatment at present (2, 3). At least 60% of the overall breast cancer among the AYA group is stage II, and above with highly associated with hormonal receptor-positive and high-grade variant. Thus, they highly likely require cytotoxic chemotherapy with prolonged endocrine therapy, which leads to low fecundity (4). Therefore, the FP treatment among AYA group is paramount. The types of cryopreservation among breast cancer varies in accordance with women's preference, marital status, and the availability of the interval timeframe for FP treatment, as suggested in the FP for breast cancer referral workflow (Figure 1). Embryo cryopreservation is an established method worldwide (5). It is cost-effective and it reduces the interval of time for pregnancy because the embryo is ready to be transferred once pregnancy is desired. However, it is only permitted for women with a steady partner or those legally married, as elaborated in our second case. This treatment is the best choice among YA women as the majority had an established relationship in their life.

Oocyte cryopreservation is also recommended as the first line of treatment among single YA women, because it was no longer considered as experimental starting from 2013 (5, 6). However, the number of oocytes is a key to determine the prediction of successful pregnancy concerning age. Among YA women, at least 15 oocytes are required to predict a 50% chance of successful pregnancy (6). Therefore, repeat COS is needed to ensure that a justified number of oocytes could be cryopreserved prior to chemotherapy. Likewise, in our first case, the patient already managed to secure 10 oocytes and continued to collect more oocytes in a given timeframe to ensure good pregnancy outcome in the future. Impromptu initiation of COS and its safety among breast cancer had been overcome by the implementation of random start (RS) protocol with a combination of AI in supplementing conventional COS. The usage of AI is well-known to stabilize the estradiol level to reduce the risk of activating estrogen-driven cancer cell (7–10). Meanwhile, the RS protocol helps overcome the delay in starting COS in the follicular phase, thus reducing the waiting time for FP treatment (10, 11). Both of these measures have been proven as an effective strategy among cancer women without jeopardizing the numbers and quality of oocytes compared with conventional COS (8). Therefore, in our first case, we managed initiating the COS and RS-AI, and she successfully cryopreserved 10 oocytes. The subsequent cycles are still ongoing, with the aim of more oocytes to be cryopreserved.

OTC is not considered as the first line for YA women with breast cancer. It is usually reserved for women with limited timeframe, because FP treatment is squeezed prior to early chemotherapy schedule or in between ongoing chemotherapy cycle, thus making the combination of oocyte and embryo cryopreservation impossible. To date, OTC is still considered as experimental (12–14). The selection of OTC candidates is currently based on Edinburg's criteria to ensure a good outcome (Table 2). Majority of the YA group who selected for FP treatment required cytotoxic chemotherapy, followed by long term hormonal suppression treatment, mainly TAM (1, 11, 12). By the time when ovarian tissue transplantation (OTT) is the aim, most of the women already had a low fertility potential due to the nature of the aging process. Fortunately, in women who received OTC, the possibility of harvesting immature oocytes simultaneously during the procedure could allow enhanced pregnancy outcome. The implementation of *in-vitro* maturation (IVM) made the maturation process possible, thus improving the FP outcome, because we managed to combine both oocytes and OTC (6, 14). This scenario was reflected in our third case, where the patient received OTC due to the limited timeframe and managed to secure 13 oocytes via IVM during the procedure.

**Table 2:** The Edinburg criteria for ovarian tissue cryopreservation (1, 11, 12).

**Criteria's**
Age < 35 years old
No previous chemotherapy/ radiotherapy if age > 15 years at diagnosis but mild, non-gonadotoxic chemotherapy if < 15 years old is acceptable
A realistic chance of surviving 5 years
A high risk of premature ovarian insufficiency (> 50%)
Inform consent (parent where appropriate)
Negative HIV, syphilis and hepatitis serology
Non-pregnant and no existing children

By contrast, the implementation of gonadotropin-releasing hormone analog (GnRHa) as gonadal protection among women with breast cancer is still inconclusive (15). Most centers utilize it as a shield to reduce the direct effect of chemotherapy by creating a transient resting follicle environment. Theatrically, the resting follicles are more resistant to chemotherapeutic agents, thereby reducing the risk of ovarian damage. Surprisingly, most of the data concluded that the level of GnRHa suppression was not sufficient to protect the ovarian tissue from chemotherapeutic damage. Therefore, most of the international bodies do not recommend the usage of GnRHa as one of the FP strategies (15, 16). Currently, GnRHa is mainly used to create the transient amenorrhea period to reduce the risk of heavy menstrual bleeding while on treatment compared with FP treatment. However, GnRHa is reserved as an FP option in centers where the established FP options, such as embryo, oocyte, or ovarian cryopreservation, were not available. Therefore, GnRHa was not offered for any of our cases (11, 15, 16).

Concerning germline mutation, at least 10% of young women with breast cancer were related to BRCA1 or BRCA2 gene. An increased risk of BC was seen in BRCA1 (20%) and BRCA2 (10%). Implementing these gene screenings has become an excellent strategy (17). However, they are still not widely available due to cost. Evidence did show that the BRCA-related BC ovarian reserve was lower than the non-BRCA BC (11, 18). Therefore, FP should be offered before primary cancer treatment. The choice of FP should be carefully discussed as OTC may have the risk of reintroducing ovarian cancer (OC) following OTT. The risk of OC is 40% for BRCA1 compared to that for BRCA2 at 15% (17). Therefore, a lengthy discussion should be offered before OTC. Furthermore, following OTC, BRCA mutation BC has a lower number of oocytes per ovarian tissue piece than non-BRCA BC, leading to lower pregnancy chance following OTT. Thus, oocyte and embryo cryopreservation is considered as a better option in terms of inadequate FP timeframe for stimulation. The use of COS-AI is also recommended to reduce the risk of breast tissue stimulation (7, 9). However, the risk of transferring to the offspring needs to be highlighted as an autosomal dominant (AD) link (17, 18). It could be carried in cryopreserved oocytes or embryos. Thus, pre-implantation genetic testing (PGT) should be offered before embryo transfer or the usage of oocytes to determine the status of BRCA mutation (18, 19). PGT could be a good FP strategy among BRCA BC women aiming for healthy offspring. The current limitation is the awareness and cost of testing that both lead to low uptake of FP among BRCA BC women. In our cases, none of the women was offered BRCA mutation screening due to cost, because it was not covered by insurance. As a proper FP strategy, BRCA mutation screening should be offered to young women with breast cancer to ensure good FP outcome.

In addition, most of the single women among breast cancer survivors in the YA group who received FP treatment have an increased possibility to remain single upon completion of the treatment (20). Therefore, the indefinite allowable duration of cryopreservation therapy and its effect on pregnancy outcome are still inconclusive. Previously published literature concluded that the duration of storage does not influence pregnancy outcome in cryopreserved material; thus, no timeframe was currently allocated for the duration of cryopreservation (21). The scenario is applicable to our first and third cases, as the cryopreservation will be renewed until they embark in a stable relationship and had a desire to conceive. Meanwhile, pregnancy in women with breast cancer was proven to be safe with no additional risk of recurrence with potentially more favorable prognosis compared with non-pregnant women with breast cancer (22, 23). However, the timing of pregnancy is essential. To date, no evidence that recommends a proper timeframe from the diagnosis to pregnancy could be found. Most of the centers depend on the molecular subtype, histological grade, and stage of cancer when anticipating the risk of recurrence following pregnancy. The first 2–3 years is mostly vital to ensure no pregnancy due to an increased risk of recurrence, particularly in estrogen receptor-positive cases. Some of the centers tend to defer up to 5 years, especially in luminal-type and in women with positive lymph nodes, to ensure no late relapse (24). Regarding the subtype of BC with FP outcome, triple-negative BC was reported to have decreased oocyte yield and pregnancy rates (19). Therefore, the urgency of FP should be highlighted to this group to ensure that an adequate number of oocytes or embryos could be cryopreserved before chemotherapy. Our triple-negative case opted for OTC due to limited time for stimulation. Fortunately, with the IVM, she was able to secure 13 MII oocytes. Thus, the combination of OTC and OC improved her future fertility outcome.

In our center, we practice a conjoint decision with breast oncologist in determining the overall patient status before allowing pregnancy. On the basis of standard rules, most of our cases are considered for conception at least after 24 months of endocrine therapy with no evidence of relapse. Currently, our center is included in the clinical trial for “Pregnancy Outcome and Safety of Interrupting Therapy for Women with Endocrine Responsive Breast Cancer” (The POSITIVE Trial, NCT02308085). This trial is recruiting women from the YA group with breast cancer who are willing to embark in pregnancy after receiving adjuvant endocrine therapy, either selective estrogen receptor modulator (SERM) alone or GnRHa + SERM or AI for ≥ 18 months but ≤ 30 months for early breast cancer (25). The study completed its first phase of recruitment and is now waiting for the analysis of the results. The estimated time for the completion of study recruitment is December 2028. Therefore, we allowed our fourth case to proceed with FET as she already finished the 2 years of TAM and deferred FET for at three 3 months following the last dose of TAM for “wash-out” period to ensure a good pregnancy outcome.

## Conclusion

Management of breast cancer women in the YA group is complex, from the variant of molecular cancer subtype to the requirement of cytotoxic chemotherapy and desire for fertility. Therefore, a proper selection of cryopreservation type and targeted timeframe for pregnancy based on a joint decision from oncofertility specialist and breast oncologist is needed to facilitate the FP treatment. To date, the selection of oocytes, embryos, and ovarian tissue is widely available as an FP treatment. However, the risk of BRCA mutation transmission should be considered among BRCA BC women, and PGT should cooperate to ensure enhanced FP outcomes. GnRHa has no gonadoprotective effect and thus should not be considered as an FP option.

## Data Availability Statement

The datasets presented in this article are not readily available because there are no data for this case series. Requests to access the datasets should be directed to nao@marianna-u.ac.jp.

## Ethics Statement

Written informed consent was obtained from the individual(s) for the publication of any potentially identifiable images or data included in this article.

## Author Contributions

YS, MA, YS-T, and NS: conceptualization. YS, MA, YH-O, ST, SS, HI, ES, and YS-T: data curation. YS, MA, YH-O, YS-T, and ST: formal analysis. YS, MA, YH-O, YS-T, ST, and NS: methodology and project administration. YH-O, YS-T, ST, and NS: supervision. ES, MA, and YS-T: writing—original draft. YS, MA, YH-O, YS-T, ST, and NS: writing—review and editing. All authors have read and agreed to the published version of the manuscript.

## Conflict of Interest

The authors declare that the research was conducted in the absence of any commercial or financial relationships that could be construed as a potential conflict of interest.

## Publisher's Note

All claims expressed in this article are solely those of the authors and do not necessarily represent those of their affiliated organizations, or those of the publisher, the editors and the reviewers. Any product that may be evaluated in this article, or claim that may be made by its manufacturer, is not guaranteed or endorsed by the publisher.
